# 

**Published:** 1865-02

**Authors:** 


*Contents.*

ORIGINAL COMMUNICATIONS........................................ 1

1 Surgical Cases of Interest, treated at Institute Hospital, Atlanta, Ga., May and June, 1864, by Surgeon D. C. O'Keefe. 2. Four Cases of Cerebro-Spinal Meningitis, reported by P. Gervais Robinson, Surgeon P. A. C. S. 3. The Value of Opium in Inflammation, especially when arising from severe Gun-shot wounds, by Joel Hall, P. A. C. S. 4. Femoral Artery Severed and not Ligated, reported by Assistant Surgeon A. C. Fox.

EDITORIAL AND MISCELLANEOUS........................................ 36

The Medical Profession. Congress of Geneva. Dyspepsia. Surgeon-General of the Federal Army.

OBITUARIES,........................................ 39

Professor James H. Conway. Surgeon St. George Peachy.

CHRONICLE OF MEDICAL SCIENCE........................................ 40

Treatment of Pneumonia and Pleurisy; being the substance of Clinical Lectures delivered in St. Mary's Hospital, London, by Thomas K. Chambers, M. D., Physician to the Hospital. On the Value of Urinary Analysis in the Diagnosis and Treatment of Hepatic Diseases, by George Harley, M. D., Professor in the University College, London. On Extraction of Soft Cataract by Suction, by T. Pridgin Teale, Jr., Surgeon to Leed's General Infirmary. On Glaucomatous Affections, and their Treatment by Iridectomy, by W. Bowman, Esq., F. R. S. Employment of Glycerine in the Treatment of Diseases of the Eye, by W. Abbotts Smith M. D The Submaxillary Ganglion. Arsenic and Quinine in Intermittent. Supplementary System of Nutrient Arteries for the Lungs. Prolapsus of the Rectum in Children. Rubidium. Woman with Three Breasts. Winter Cherry. Daturine.

